# Links between Sleep Apnoea and Insomnia in a British Cohort

**DOI:** 10.3390/clockssleep5030036

**Published:** 2023-09-11

**Authors:** Yizhou Yu

**Affiliations:** 1 International Sleep Charity, Shedfield, Southampton SO32 2HN, UK; yizhou0421@gmail.com; 2 MRC Toxicology Unit, University of Cambridge, Tennis Court Road, Cambridge CB2 1QR, UK

**Keywords:** insomnia, sleep apnoea, digital health, Mendelian randomisation, fatigue, prevalence, machine learning, insomnia, public health, mental health

## Abstract

Poor sleep is a major public health problem with implications for a wide range of critical health outcomes. Insomnia and sleep apnoea are the two most common causes of poor sleep, and recent studies have shown that these disorders frequently co-occur. Comorbid insomnia and sleep apnoea can substantially impair quality of life and increase the overall risk of mortality. However, the causal and physiological links between sleep apnoea and insomnia are unclear. It is also unknown whether having a higher risk for one condition can increase the risk of developing the other. Here, we investigated links between sleep apnoea and insomnia in a British population using a combination of self-reported questionnaires and causal inference. We found that 54.3% of the cohort had moderate insomnia, 9.4% had moderate sleep apnoea, and that 6.2% scored high for both conditions. Importantly, having a higher risk of sleep apnoea was associated with a higher risk of insomnia and vice versa. To determine the causal directionality between sleep apnoea and insomnia, we used Mendelian randomisation and found evidence that sleep apnoea could cause insomnia, but not the reverse. To elucidate how both sleep apnoea and insomnia were linked to each other, we looked at the behavioural markers of poor sleep. We found that feeling fatigued after sleeping and having noticeable sleep problems were linked to a higher burden of both sleep apnoea and insomnia. In conclusion, our results show that sleep apnoea increases the risk of developing insomnia, and both conditions can result in fatigue. We highlight the importance of considering and treating the symptoms of both conditions.

## 1. Introduction

Poor sleep is a major health, economic, and societal burden [1]. Disturbed sleep can negatively impact mood, cognitive performance, and quality of life [2]. Sleep disturbances are common in neurological diseases [3] and the prevalence of sleep-related problems increases with age [4]. With an ageing population, understanding the causes of poor sleep represents an important public health question.

The most prevalent causes of poor sleep are sleep apnoea and insomnia. Sleep apnoea refers to the cessation of breathing during sleep. The most common form of sleep apnoea is called obstructive sleep apnoea (OSA), which is the cessation or reduction of breathing during sleep despite a continued ventilatory effort. It is clinically defined by the Apnea-Hypopnea Index (AHI), which is calculated as the number of pauses in breathing (apnoea) and periods of shallow breathing (hypopnea) per hour. OSA is linked to lower blood oxygen levels and higher carbon dioxide levels, which lead to frequent awakenings and difficulties falling asleep. Sleep fragmentation can cause neurological and cardiovascular damage [5,6,7]. Conversely, insomnia is often characterised by difficulties initiating and/or maintaining sleep, resulting in daytime functional impairment [8]. Stress, anxiety, taking sleep-related medications, and having poor sleep hygiene can also influence insomnia risk [9].

Poor sleep has both short- and long-term physiological impacts [10]. Sustained sleep disturbance resulting from sleep apnoea [11,12] and insomnia [13,14] has been associated with cognitive deficits, neurodegeneration, and mental health issues [15]. Recent reports on the prevalence of poor sleep in adults show that more than 33% of the population experiences poor sleep [16,17,18]. In countries including China, the United States of America, Brazil, and India, the prevalence of OSA exceeded more than 50% of the population, and a majority of sufferers remain untreated [12]. Importantly, this number has been steadily increasing [19], clearly indicating a need to detect signs of poor sleep in the general population and promote better sleep hygiene.

These two major types of sleep disturbances can co-occur and are referred to as comorbid insomnia and sleep apnoea (COMISA) [20]. One of the pioneering reports on COMISA was published in 1973 [21]. Since then, studies have reported a prevalence of COMISA from 10% to 50% [20,21,22,23,24,25]. Importantly, having COMISA increases the risk of developing cardiovascular diseases and causes a 47% increase in mortality compared to individuals without sleep conditions [26,27,28].

Questionnaires are common diagnostic tools used in primary care because they provide a quantitative measure of the subjective quality of sleep. Sleep questionnaires such as STOP-Bang, the Multivariate Apnoea Prediction Index (MAPI), and the Insomnia Severity Index (ISI) have been validated repeatedly and shown to correlate with clinical symptoms and objective testing [29,30,31,32]. Each question from these questionnaires was developed to address particular features of the specific sleep disorder. However, the way in which they might correlate with each other in the case of COMISA burden is unclear.

In this study, we investigated links between sleep apnoea and insomnia by combining an observational method with Mendelian randomisation. First, we found that when using questionnaire-based methods, the prevalence of moderate insomnia and sleep apnoea is 54.3% and 9.4%, corroborating previous studies [33]. We add that the prevalence of COMISA in our cohort was 6.2%. Using Mendelian randomisation, we showed that sleep apnoea could cause insomnia. We then asked what self-reported markers of poor sleep are common between individuals with high risks of having sleep apnoea and insomnia. Using questionnaires, we found that feeling fatigued after sleeping and having noticeable sleep disturbances are common features of both sleep apnoea and insomnia. We conclude that an integrated approach that screens for both conditions as a general feature of poor sleep is required.

## 2. Methods

### 2.1. Data Collection

The data was collected by SleepHubs Limited (a company registered in England under GB10520947) during an 8-month period from September 2019 to April 2020. An online questionnaire hosted on the SleepHubs website containing the STOP-Bang questionnaire, the ISI, the MAPI, and the SleepHubs Check-Up was answered by 1198 healthy adults from the United Kingdom. Individuals explicitly consented to the collection and use of anonymised data and were aware that their data was collected for research. Should any anxiety arise from answering the questionnaire, links to professional services were made available at the end of the questionnaire. All data collection methods conform to the Declaration of Helsinki.

This paper was a secondary data analysis of robustly anonymised data with minimal demographic information collected, where there is no chance of data being linked to any individuals. According to the NHS Health Research Authority, ethical approval was not required for this research.

### 2.2. Sleep-Related Questionnaires

Data from the STOP-Bang questionnaire, the ISI, and the MAPI were collected and scored according to their published scoring calculations [29,34,35,36]. The choice of these questionnaires was based on their validated accuracy. A meta-analysis of 26,547 participants showed that the accuracy of the STOP-Bang questionnaire is above 90% [37]. Similarly, a meta-analysis of 4693 participants showed that the Insomnia Severity Index has a sensitivity and specificity of 88% and 85%, respectively [38]. The questions used in the SHC are adapted from the Screen for Sleep Disorders [39]. Questions were chosen for their brevity, simplicity, and link between multiple sleep conditions. There are four main questions: “How do you feel during the day?” (abbreviated to “Feeling sleepy during the day”); “Are you happy with your sleep?”; “Do you ‘nod off,’ doze, or fall asleep during the day without obvious reason?”; and “Has anyone ever complained about your sleep?”. Visit https://sleephubs.com/ for the full questionnaire (accessed on 20 August 2019).

### 2.3. Statistical Analysis

The raw data was first scaled by normalising the data by the mean and standard deviation. Multivariate linear models and principal component analysis were performed using the programming language R, version 4.0.0, as described in our previous work [40,41,42,43]. The plots were generated using yy_tools (https://github.com/izu0421/yytools). All code is made available on our GitHub repository (https://github.com/izu0421/osa_insomnia or https://www.yizhouyu.com/osa_insomnia/).

### 2.4. Mendelian Randomisation

To investigate the causal links between insomnia and sleep apnoea, we used Mendelian randomisation, as previously described [44]. Summary statistics of genome-wide association studies (GWAS) for insomnia were obtained from the UK Biobank [45] and the corresponding data for sleep apnoea were obtained from FinnGen [46]. FinnGen is a large-scale research project in Finland that aims to gather and analyse genetic data from over 500,000 Finnish individuals to improve the understanding of the relationship between genetics, lifestyle, and diseases. To recruit participants, FinnGen has partnered with several healthcare organisations in Finland, such as public and private hospitals as well as legacy samples from Finnish biobanks. The UK Biobank recruited over 500,000 participants aged between 40 and 69 years across the UK between 2006 and 2010. Recruitment involved inviting individuals from the general population who were registered with the National Health Service and lived close to an assessment centre. Potential participants received invitations by mail and were required to respond to a number of questionnaires, provide samples for genetic analysis, and undergo a variety of physical examinations and assessments.

MR analysis involves 3 main steps [47]: Step 1 involves the selection of genetic variants associated with the exposure of interest which, in this case, is the summary statistics from GWAS. These genetic variants mimic natural experiments, as they are inherited randomly and are not influenced by confounding factors. In step 2, these selected genetic variants are examined to determine their effects on the outcome, like sleep apnoea for example. This step simulates the design of a randomised controlled trial, using genetic data as a proxy for randomisation. In step 3, comparing the effects of the genetic variants on the exposure (e.g., insomnia) and the outcome (e.g., sleep apnoea) allows us to estimate the extent to which insomnia might cause sleep apnoea, for example.

Both of these datasets are available on our GitHub repository (https://github.com/izu0421/osa_insomnia). The genetic data of these two datasets were harmonised using the MR-Base packages [48] on R 4.0.0. We used the inverse-variance weighted method to obtain the combined effect size due to its robustness and reliability [49]. The harmonised datasets, code, and formulae used to reproduce our results are available on our GitHub repository (https://www.yizhouyu.com/osa_insomnia/).

## 3. Results

### 3.1. Prevalence of Sleep Apnoea, Insomnia, and COMISA in a British Population

**Figure 1:**
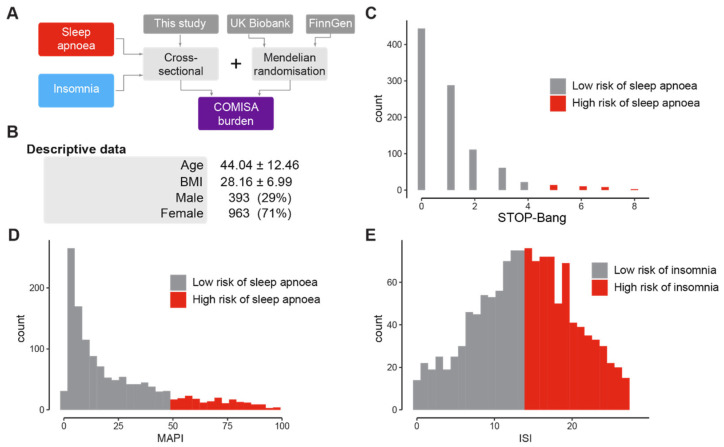
**Prevalence of poor sleep in a UK cohort.** (**A**). Overall workflow of the analysis. We first investigated the co-occurrence of sleep apnoea and insomnia using questionnaires from a cross-sectional cohort. We then complemented this analysis using Mendelian randomisation, which mimics a clinical trial using genetics, by combining GWAS summary statistics from the UK Biobank and FinnGen. Finally, we investigated behavioural factors that could be common to both conditions. The sleep disturbances investigated are in red (sleep apnoea risk) and blue (insomnia risk). The sources of the datasets are in dark grey. The methods used are in light grey. The main target of this investigation, COMISA, is in purple. (**B**). Descriptive statistics of the cohort. For the “Age” and “Body Mass Index” (BMI) categories, the mean and standard deviation are shown. For the “Gender” category, the number and percentage are shown. (**C**–**E**). Distribution of the STOP-Bang (**C**), MAPI (**D**), and ISI (**E**) scores. A STOP-Bang score above 4 is considered high risk for sleep apnoea [34]. Having a MAPI score above 50 is linked to a high risk of sleep apnoea [35]. An ISI score above 14 is considered high risk for moderate insomnia [29,31].

To investigate features of poor sleep, we collected self-reported sleep data from an adult cohort in the United Kingdom (see Figure 1A for overall workflow). Our cohort was composed of predominantly female participants with an average age of 44 years and an average BMI of 28 (Figure 1B). Since sleep apnoea and insomnia are the most prevalent causes of poor sleep, we analysed questionnaires that reported the risk of sleep apnoea and insomnia. We used the STOP-Bang and MAPI questionnaires to measure sleep apnoea burden (Figure 1C and D respectively), and the ISI questionnaire to quantify insomnia burden (Figure 1E).

To assess whether the general characteristics of our population are similar to that of other cohorts, we calculated the prevalence of insomnia and sleep apnoea and compared them to those from previous research. We found that 54.3% of our cohort scored above 14 on the ISI questionnaire, which indicates a moderate risk of insomnia. This prevalence is similar to that of a large meta-analysis of 48 studies over a similar period [50], indicating that the prevalence of insomnia reflects published literature. Conversely, we found that 9.4% of the cohort scored high on either the MAPI or STOP-Bang questionnaires for moderate sleep apnoea. This prevalence is similar to that of a global estimation and a clinical study [12,51]. Taken together, these results indicate that the prevalence of sleep apnoea and insomnia observed in our questionnaire-based study are similar to results from objective measurements.

**Figure 2:**
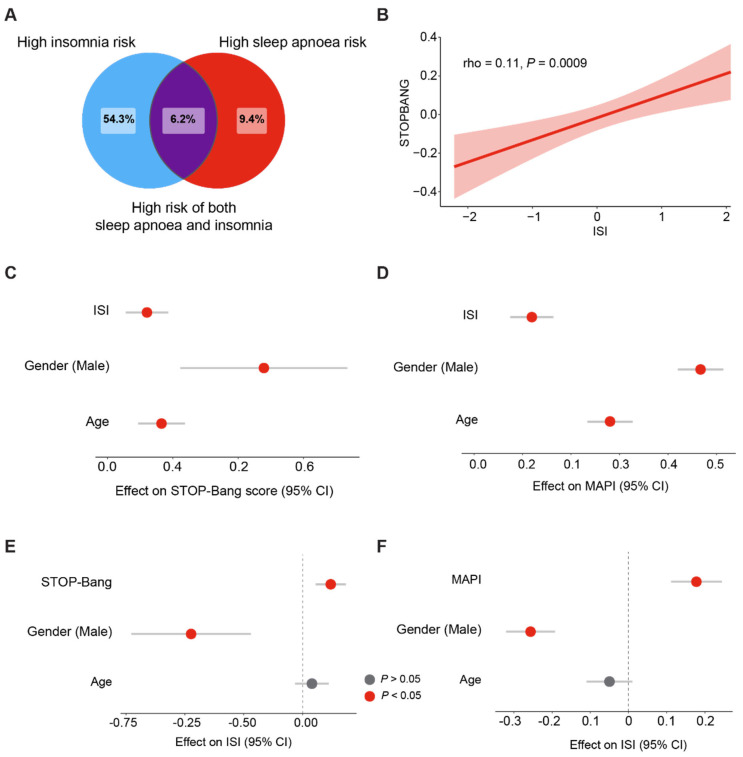
**Links between self-reported sleep apnoea and insomnia scores.** (**A**). Prevalence of high levels of both insomnia and sleep apnoea. Values represent the percentage of participants. (**B**). Correlation between the ISI and STOP-Bang scores (Spearman’s rank correlation, rho = 0.11, *p* = 0.0009). (**C**). Effect of the ISI on the STOP-Bang score (Linear regression, adjusted R^2^ = 0.05, *p* < 0.0001). (**D**). Effect of the ISI on the MAPI (Linear regression, adjusted R^2^ = 0.37, *p* < 0.0001). (**E**). Effect of the STOP-Bang score on the ISI (Linear regression, adjusted R^2^ = 0.02, *p* < 0.0001). (**F**). Effect of the MAPI on the ISI (Linear regression, adjusted R^2^ = 0.05, *p* < 0.0001). For ease of comparison, all values are scaled by subtracting the mean and dividing by the standard deviation. Gender is represented as male, compared to female.

We next asked what the prevalence was of having a high risk for both sleep apnoea and insomnia. Strikingly, we found that 6.2% of our cohort had COMISA (Figure 1A). This indicates that participants with moderate sleep apnoea are approximately 66% more likely to also have moderate insomnia. Given the high prevalence of both conditions, we tested whether having one condition would increase the risk of having the other. First, we found a positive and statistically significant correlation between sleep apnoea burden and insomnia burden (Figure 2B). To assess the robustness of our analyses, we used regression models and found that a high insomnia burden is linked to a higher risk of having sleep apnoea (Figure 2C,D), after adjusting for age and sex as confounding variables. Similarly, we found a positive relationship between sleep apnoea and insomnia (Figure 2E,F). Therefore, we conclude that having either insomnia or sleep apnoea is associated with an increased risk of also having the other condition.

### 3.2. Sleep Apnoea Is a Putative Cause of Insomnia

**Figure 3:**
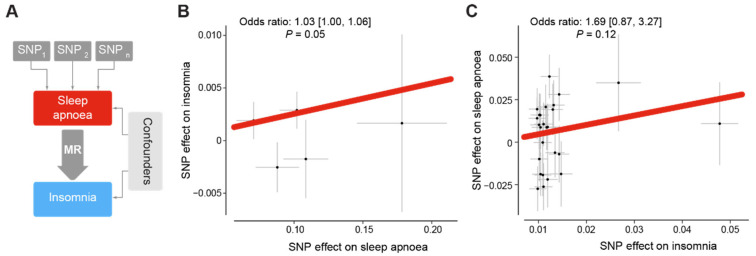
**Sleep apnoea is causally linked to an increased risk of insomnia.** (**A**). Two-sample Mendelian randomisation (MR) assesses causal relationships between an exposure (one sleep disorder, e.g., sleep apnoea) and an outcome (another sleep disorder, e.g., insomnia). It leverages genetic variants that are associated with the exposure of interest to infer causality. Since the genetic variants are associated with the exposure but not influenced by confounders related to the outcome, the inferred causal relationship becomes more reliable. (**B**). MR analysis of the effect of sleep apnoea on insomnia. (**C**). Reverse analysis of the effect of insomnia on the odds of developing sleep apnoea. For (**B**,**C**), the odds ratios, 95% confidence intervals, and *p* values are shown. Weighted median models were used. Each dot represent an SNP and the grey lines represent the standard errors of the effects of the SNP.

Given the strong link between sleep apnoea and insomnia that we observed, we next investigated whether sleep apnoea can cause insomnia and vice versa. Mendelian randomisation (MR) uses natural variations in genes to mimic a clinical trial (Figure 3A). MR has been used to determine the causal link between diseases, while overcoming technical and ethical constraints [47]. Previous research remarked that respiratory events caused by sleep apnoea can lead to nocturnal awakenings and insomnia-like symptoms [52]. Therefore, we used MR to test whether sleep apnoea can cause insomnia. We combined genome-wide statistics on insomnia from a cohort totalling 336,965 individuals from the UK Biobank [45] as well as genetic correlates for sleep apnoea from a cohort of 217,955 participants from the FinnGen consortium [46]. We found that having a high risk of sleep apnoea significantly increases the odds of developing insomnia by approximately 3% (Figure 3B). Using the same two cohorts, we tested whether insomnia can also cause sleep apnoea. We found a positive link between insomnia and sleep apnoea, but this link is not significant (Figure 3C). Taken together, we found evidence that sleep apnoea could cause insomnia, but no strong evidence for the reverse.

### 3.3. Fatigue and the Noticeability of Sleep Problems Are Markers of Poor Sleep

**Figure 4:**
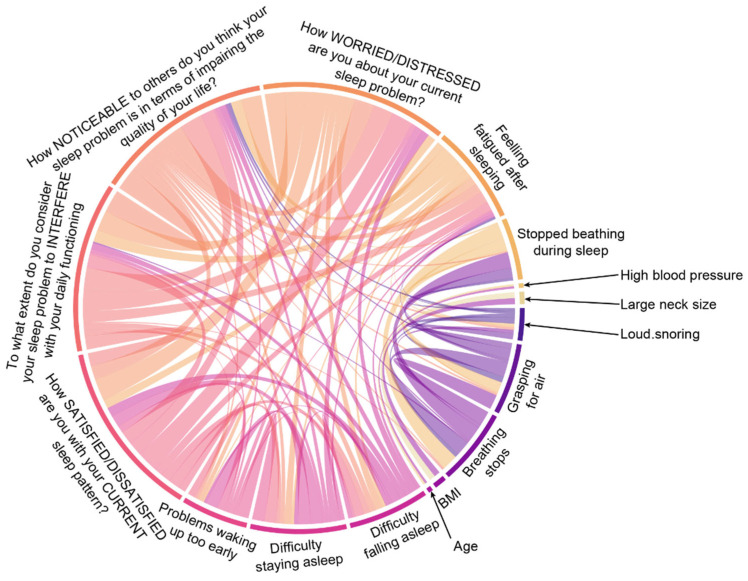
**Correlations between markers of insomnia and sleep apnoea.** Significantly correlated variables as determined using the Spearman’s rank test are shown. The false discovery rate was controlled using the Benjamini–Hochberg method. Only significantly correlated variables are shown. Each colour represents a different question from the sleep questionnaires used in this study. The thickness of each string connecting 2 variables is proportional to the strength of the correlation (correlation coefficient). The names of the variables were shortened for ease of display.

**Figure 5:**
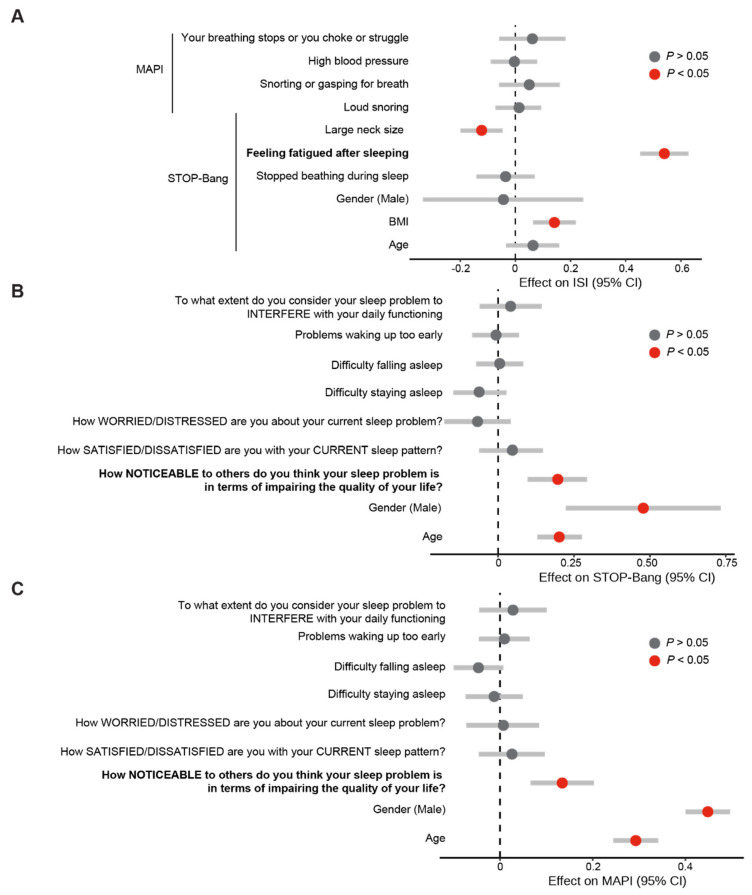
**Effect of markers of sleep apnoea on insomnia and vice versa.** (**A**). Effect of markers of sleep apnoea on insomnia (Linear regression, adjusted R^2^ = 0.31, *p* < 0.0001). (**B**). Effect of markers of insomnia on sleep apnoea as measured by the STOP-Bang questionnaire ((**B**), Linear regression, adjusted R^2^ = 0.38, *p* < 0.0001) and the MAPI ((**C**), linear regression, adjusted R^2^ = 0.38, *p* < 0.0001). Statistically significant variables are indicated in red and non-significant variables are in grey. The investigated variables are in bold.

Our previous results highlighted the co-occurrence of these sleep disorders and the causal link between sleep apnoea and insomnia. However, it is unclear which aspect of sleep apnoea is linked to a higher risk of insomnia. Therefore, we sought to investigate what component of the sleep apnoea questionnaires is linked to insomnia and vice versa. We first explored whether self-reported markers of poor sleep correlate with each other. Using a pair-wise comparison with adjustment for false discovery, we found that markers of insomnia correlate with each other, as well as with markers of sleep apnoea (Figure 4). Given these associations, we investigated whether any questions from sleep apnoea questionnaires can predict insomnia burden. Using a regression model, we found that feeling fatigued after sleeping is significantly associated with a higher burden of insomnia (Figure 5A). Using the same analysis to investigate whether markers of insomnia are linked to sleep apnoea burden, we found that the noticeability of sleep problems is linked to a higher risk of sleep apnoea in both the STOP-Bang (Figure 5B) and MAPI questionnaires (Figure 5C).

**Figure 6:**
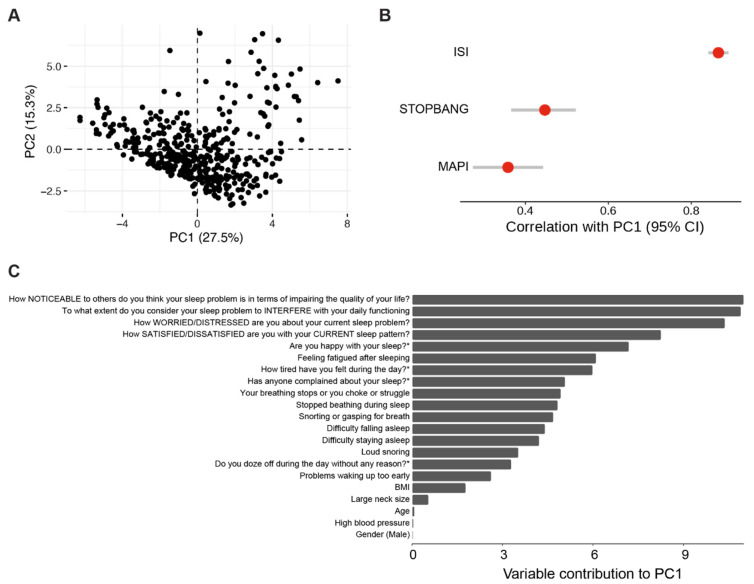
**General features of poor sleep.** (**A**). Principal component analysis of all markers of poor sleep. Each dot represents a participant. (**B**). Correlation between the first principal component and indices of poor sleep (Pearson’s product-moment correlation, *p* < 0.0001 for all comparisons). Each red dot represents the correlation coefficient and the grey bars represent the confidence intervals. (**C**). Feature loading of the first principal component. The 4 questions from the SleepHubs Check-Up are labelled with an asterisk.

Since we found links between sleep apnoea and insomnia at the genetic and behavioural levels, we reasoned that there could be commonalities of poor sleep within these conditions. Each question from sleep-related questionnaires might capture a certain extent of information on poor sleep. We therefore asked whether the two markers of poor sleep, fatigued after sleeping and noticeable sleep problems, contribute to poor sleep in general. To measure poor sleep as an unobserved variable, we used principal component analysis (PCA) to perform feature reduction on all of the available questions in our dataset. Those questions are from the sleep apnoea and insomnia questionnaires and also include four additional questions from the SleepHubs platform (SleepHubs Check-Up). Our PCA shows that the first principal component (PC1) captures 27.5% of the variance (Figure 6A). This indicates that PC1 captured underlying information on sleep apnoea and insomnia. We tested this assumption by investigating whether the ISI, MAPI, and STOP-Bang score are associated with PC1, and found that all of them are significantly associated with PC1 (Figure 6B). This further confirms that PC1 is likely to have captured underlying information on both sleep apnoea and insomnia. We therefore asked what questions from the questionnaires contributed the highest to PC1. We analysed the contributions of PC1 and found that the noticeability of sleep problems is indeed the largest contributor to poor sleep, as modeled by PC1 (Figure 6C). From the questions belonging to the MAPI and STOP-Bang questionnaires, fatigue after sleeping was the largest contributor to poor sleep. Taken together, these analyses show that fatigue and noticeability of sleep problems correlate with poor sleep.

### 3.4. Validation of Fatigue and Noticeability of Sleep Problems as Reproducible Markers of Poor Sleep Using the SleepHubs Check-Up

**Figure 7:**
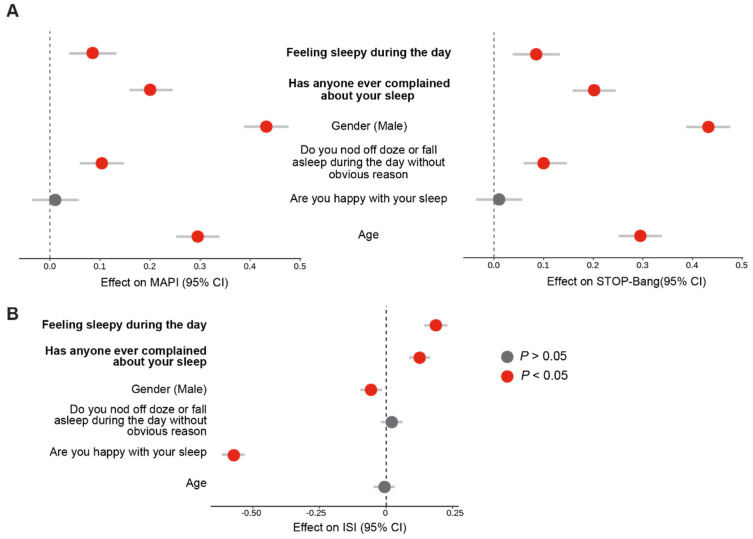
**Measurement of poor sleep using the SleepHubs Check-Up.** (**A**). Correlation between questions from the SleepHubs Check-Up and markers of sleep apnoea from the MAPI on the right (Linear regression, adjusted R^2^ = 0.43, *p* < 0.0001) and STOP-Bang on the left (Linear regression, adjusted R^2^ = 0.43, *p* < 0.0001). (**B**). Links between questions from the SleepHubs Check-Up and ISI (Linear regression, adjusted R^2^ = 0.54, *p* < 0.0001). Statistically significant variables are indicated in red and non-significant variables are in grey. The investigated variables are in bold.

A common issue in characterising health problems using questionnaires is phrasing, which can contribute to bias [53]. It is conceivable that the wording of a question can affect how a participant understands and answers that question. We previously identified that fatigue and noticeability of sleep problems correlate with both insomnia, sleep apnoea, and a compound variable that correlates with poor sleep. In order to assess whether these findings are robust and reproducible, we asked whether small changes in these questions could change the results. The SleepHubs Check-Up is a short questionnaire that comprises four main questions. Of these, one asks about how sleepy a participant feels during the day and another asks if anyone complained about their sleep. These two questions respectively allude to fatigue and the noticeability of sleep problems. Therefore, we investigated whether questions from the SleepHubs Check-Up are predictive of sleep apnoea and insomnia. Our results confirmed that both feeling sleepy during the day and receiving complaints about sleep are significantly correlated with a higher burden of sleep apnoea (Figure 7A) and insomnia (Figure 7B). We therefore concluded that feeling sleepy during the day and noticeable sleep problems are robust predictors of poor sleep.

## 4. Discussion

There is a high prevalence of undiagnosed insomnia and OSA [54,55,56,57,58], representing a major public health challenge. Additionally, recent research found a high prevalence of OSA in insomnia patients and vice versa [22,57], indicating the need for better and more efficient tools to understand insomnia, OSA, and COMISA in the general population [26].

The aim of our study was to understand the cause and phenotypic features of people with a high risk of developing insomnia and sleep apnoea. We found a high prevalence of people with moderate insomnia risk (54.3%), sleep apnoea risk (9.4%), and both conditions (6.2%). Since we found a potential co-prevalence between sleep apnoea and insomnia risks, we sought to investigate whether having one could lead to the other. Mendelian randomisation is a statistical method that uses genetic variations to investigate the causal relationship between a certain exposure and an outcome. In this case, the exposure is sleep apnoea and the outcome is insomnia. By using genetic data, which is not influenced by environmental or behavioural factors, Mendelian randomisation can provide stronger evidence of causality compared to traditional observational studies. Using this method, we found evidence that sleep apnoea can cause insomnia. Insights from our analysis provide a strong basis for further research into the underlying mechanisms and potential interventions for both conditions.

Previous studies found symptoms of sleep apnoea in insomnia patients [26,59]. Importantly, treating sleep apnoea alleviated symptoms related to insomnia [60]. This suggests that sleep apnoea may play a causal role in the development of insomnia. Thus, treating sleep apnoea may be effective in reducing the symptoms of insomnia in COMISA patients [26]. However, how sleep apnoea can cause insomnia is unclear. The repetitive obstructive events in sleep apnoea could cause the development of sleeplessness, a feature of insomnia, in sleep apnoea patients [33]. As a result, it is possible that patients with both an underlying burden of insomnia and sleep apnoea would feel fatigued after sleeping. Indeed, we found that feeling fatigued is robustly associated with the multiple markers of poor sleep investigated in this study. Our findings support previous observations claiming that feeling fatigued may be a consequence of poor sleep, either due to sleep apnoea or insomnia [19,59]. Some behavioural adaptations to feeling fatigued could include increased caffeine intake. Additionally, disrupted sleep, either due to sleep apnoea or insomnia, might lead to increased anxiety [61]. Anxiety can, in turn, cause insomnia, creating a pathological feedback loop [62]. It is conceivable that an individual initially diagnosed with sleep apnoea would first feel fatigued, then become more anxious, and eventually develop insomnia. Further research is needed to understand the complex relationship between sleep apnoea, insomnia, and anxiety.

Our epidemiological data showed that having noticeable sleep problems, which is a question related to insomnia, predicts a higher sleep apnoea score in both the STOP-Bang and MAPI questionnaires. Similarly, higher STOP-Bang and MAPI scores also predict a higher ISI score. However, our Mendelian randomisation-based analysis did not find any evidence to support the claim that insomnia causes sleep apnoea. A possible explanation could be the structure of the cohorts that we used. Sleep apnoea is more prevalent in people of Hispanic and Chinese ethnicities [63]. It is therefore possible that genetic variants discovered in individuals of European descent from the UK Biobank and FinnGen fail to capture important information linked to sleep apnoea and insomnia. We therefore do not exclude the possibility that insomnia can cause sleep apnoea.

Since part of this research featured an online questionnaire, we were cautious with our data analysis. For instance, there could have been a bias in the respondents whereby people with more health concerns are more likely to answer sleep-related questionnaires. Another limitation of our study is the lack of objective clinical measurements on AHI and polysomnography, which are more objective than questionnaires for diagnosing sleep apnoea and insomnia. Notably, we highlight that our results must be interpreted with caution as questionnaires quantify a risk of developing sleep apnoea or insomnia, which is different from a clinical diagnosis. More accurate and objective quantifications of sleep apnoea and insomnia are therefore required. Nonetheless, our study replicated previous findings and showed similar prevalences of the two conditions. For instance, we found that female participants and those with a higher BMI are more likely to have a higher insomnia risk [64,65]. We also found that male participants with a higher BMI and age are more likely to have a higher sleep apnoea score [66]. These observations demonstrate the validity of collecting data using an online platform and add assurance to our novel observations. It is also worth noting that the data collection period overlaps with COVID-19, which could have affected how participants answered the questionnaire. It would therefore be interesting to analyse the impact of COVID-19 on the markers of sleep quality using this dataset.

In conclusion, our study found a high prevalence of both sleep apnoea and insomnia in our cohort, and evidence of a possible causal relationship between the two conditions. Further research can use techniques like Mendelian randomisation to investigate the modifiable health factors that can cause both sleep apnoea and insomnia. A better understanding of these two conditions as a whole could improve sleep therapies and public health. Moreover, our study also highlights the validity of collecting health information through digital technologies and calls for further investigations on sleep health using digital methods.

## Data Availability

The GWAS summary data used for Mendelian randomisation is available in our repository https://github.com/izu0421/osa_insomnia. Epidemiological data can be requested from SleepHubs (sleephubs.com). All code is available on our GitHub repository https://github.com/izu0421/osa_insomnia.

## Abbreviations

ISI, insomnia severity index; MAPI, multivariable apnoea prediction index; OSA, obstructive sleep apnoea; MR, Mendelian randomisation; COMISA, comorbid insomnia and sleep apnoea; AHI, Apnea-Hypopnea Index
